# Cross-Linguistic Influences on L2 Prosody Perception: Evidence from English Interrogative Focus Perception by Mandarin Listeners

**DOI:** 10.3390/brainsci15091000

**Published:** 2025-09-16

**Authors:** Xing Liu, Xiaoxiang Chen, Chen Kuang, Fei Chen

**Affiliations:** School of Foreign Languages, Hunan University, Lushannan Road No. 2, Yuelu District, Changsha 410082, China; liuxing@hnu.edu.cn (X.L.); icprll2014@hnu.edu.cn (X.C.); kuangchen@hnu.edu.cn (C.K.)

**Keywords:** L2 prosody perception, Mandarin speakers, focus prosody, interrogative prosody

## Abstract

Background/Objectives: This study sets out to explore how L1 Mandarin speakers with varying lengths of L2 experience perceived English focus interrogative tune, L*H-H%, within the framework of the autosegmental–metrical model. Methods: Eighteen Mandarin speakers with varying lengths of residence in the United States and eighteen English native speakers were invited to perceive prosodic prominence and judge the naturalness of focus prosody tunes. Results: For the perception of on-focus pitch accent L*, Mandarin speakers performed well in the prominence detection task but not in the focus identification task. For post-focus edge tones, we found that phrase accents were more susceptible to L1 influences than boundary tones due to the varying degrees of cross-linguistic similarity between these intonational categories. The results also show that even listeners with extended L2 experience were not proficient in their perception of L2 interrogative focus tunes. Conclusions: This study reveals the advantage of considering the degree of L1-L2 similarity and the necessity to examine cross-linguistic influences on L2 perception of prosody separately in phonological and phonetic dimensions.

## 1. Introduction

As an integral part of speech prosody, pitch variations are employed to convey different meanings across world languages. For example, in intonation languages, such as English, pitch variations bear mostly post-lexical meanings, but in tone languages, like Mandarin, apart from intonational meanings, pitch changes are mainly used to distinguish lexical meanings. It is, therefore, intriguing to explore to what extent L2 learners are affected by their L1 prosodic features when learning an additional prosody system [1,2,3]. For tone language speakers, when learning an intonation language, they might be subject to even more complicated cross-linguistic influences, with L1 features from both lexical and post-lexical levels.

To understand how these cross-linguistic prosodic features influence L2 prosody development, we specifically investigated how Mandarin native speakers perceive English focus interrogative tune, L*H-H%, in the framework of the autosegmental–metrical (AM) model (see [4] for an overview). As a typical tune in Mainstream American English, L*H-H% serves a two-fold purpose. First, the tune form of L*H-H% is relatively fixed regardless of its domain size, be it aligned with one single syllable or a larger linguistic unit. The form-meaning redundancy in prosody can thus be well reduced [5]. Second, the pragmatic meaning of focus could control for the speaker’s idiosyncrasies to a large extent, which has been notoriously elusive in prosody research [6,7].

### 1.1. A Comparative Analysis of Interrogative Focus Prosody in English and Mandarin

In the AM model of intonation, utterances are organized in a hierarchical structure as phonological words, intermediate phrases, and intonation phrases [6,8,9,10]. In English, an intonation phrase is always composed of pitch accents, phrase accents, and boundary tones, which are represented as T*, T-, and T%, respectively, in the ToBI transcription system [11]. A typical English interrogative contour is represented as L*H-H% [12]. In this intonational contour, the pitch accent L* is always aligned with the focused constituent, and the rest part, including phrase tone H- and boundary tone H%, is collectively called post-focus edge tones [13]. In contrast, Mandarin has a rather different prosodic structure from English, with prosodic phrases and breath groups above the prosodic word level [14]. There are no pitch accents or phrase accents in Mandarin, and tones are densely specified by the lexicon. Boundary tones T% are employed to distinguish declarative and interrogative meanings in the same way as in English. A typical Mandarin question is always marked with a boundary tone H% [15,16].

**Figure 1:**
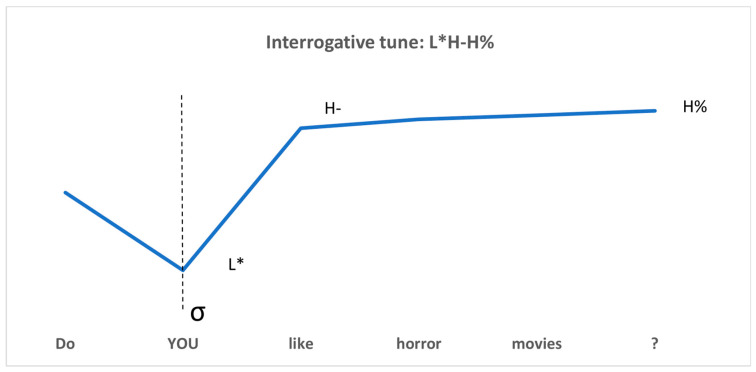
A schematic representation of English interrogative focus prosody.

As an integral part of L2 phonology, focus prosody can mark language focus meaning effectively, in addition to morphological and syntactic means. A universal tendency across world languages associates prosodic prominence with a high tone. However, English focus in interrogative environments is distinctive, since it is realized through an L* tone aligned with the focused element, followed by a high, toneless pitch plateau in the post-focus region. To convey focus meaning, the focused part is always pitch accented, and the post-focus part is deaccented and dephrased [17,18]. That is, for example, in the yes–no question “Do YOU like horror movies?”, L* is always aligned with the stressed syllable “YOU” in the focused constituent, and the post-focus parts “like horror movies” are deaccented and realized with a high pitch plateau H-H% (see Figure 1).

**Figure 2:**
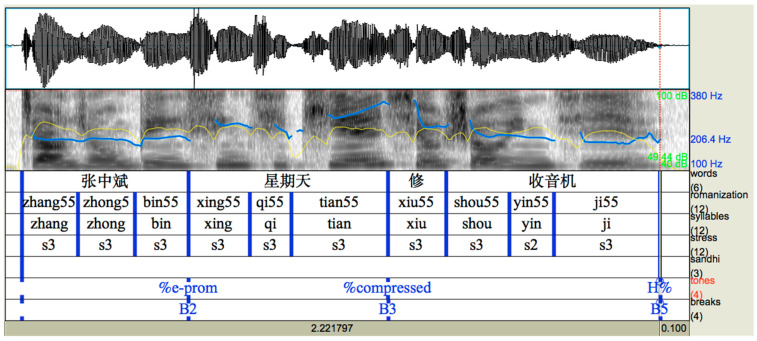
M-ToBI transcription of a Mandarin echo question “张中斌星期天修收音机?” (literal translation: Zhang Zhongbing Sunday repaired radio? Zhang Zhongbin repaired the radio on Sunday?) with sentence-medial focus “xingqi tian (Sunday)”.

Unlike in English, intonational prominence in Mandarin is conveyed through an expansion of the pitch range in the focused region and a compression of the pitch range in the post-focus region, while preserving the underlying tonal contour [15]. In terms of phonetic implementation, when on-focus syllables are realized longer and louder, their post-focus pitch contours are largely determined by the lexical tones, with pitch-range compression until the last syllable [19,20]. Specifically, in interrogative sentences, unlike the high plateau (H-) in English, the post-focus regions in Mandarin remain globally low until the final syllable, which is typically realized as a rise with the boundary tone H% (see Figure 2).

In summary, the cross-linguistic comparison between English and Mandarin interrogative focus prosody can be effectively conducted by analyzing both the phonological representations and their phonetic realizations with the AM model of intonation. More importantly, both on-focus pitch accent L* and post-focus edge tones H-H% in the interrogative intonational settings can be examined within this universal theoretical framework of intonation.

### 1.2. Cross-Linguistic Influences on L2 Interrogative Focus Prosody Development

As seen in the existing L2 speech learning models, such as the Perceptual Assimilation Model (PAM [21,22,23]) and the Speech Learning model (SLM [24,25]), the degree of cross-linguistic similarity has long been a key issue in predicting the difficulty of L2 learning. However, in comparison to segments, it is much more complicated to determine the degree of cross-linguistic similarities in speech prosody, not least because of various theoretical assumptions. By adopting the AM model of intonation, Mennen [26] proposed a working model of L2 intonation learning, the L2 Intonation Learning theory (LILt), which suggests comparing intonation systems along four dimensions: systematic, realizational, semantic, and frequency. With this framework, L2 learning difficulties in prosody can be better predicted.

So far, a few studies have been undertaken to explore how L1 prosodic features influence L2 learning of prosodic prominence. For example, Graham and Post [27] investigated the L2 English pitch accent production patterns by L1 Japanese and Spanish speakers. They examined both pitch accent patterns and pitch alignment in the systematic and realizational dimensions of LILt. Wayland et al. [28] explored how L1 Mandarin speakers perceived English focus in echo questions. However, when these studies examined the prominent parts, how L2 learners perceived the post-focus contours has been largely overlooked. To capture the details in cross-linguistic influences on the development of L2 prosody, it is necessary to examine how L2 learners perceive post-focus intonational categories, i.e., phrase tones and boundary tones, as the prosodic structure of L1 and L2 might be very different in spite of their similar phonetic realization.

However, in comparison to on-focus pitch accents, it is much more complicated to measure the perception of post-focus edge tones directly, for these tones usually serve as the background of linguistic meanings, and they are phonetically less prominent [20]. There have been very few studies on the acquisition of post-focus compression, and they have been mainly based on the phonetic realizations. Grabe et al. [29] found L1 Mandarin speakers mistakenly perceived L*H-L% as the rising intonation (LH), and this misperception was attributed to the extended F0 plateau following the focus. It remains to be determined whether L2 speakers can accurately perceive each category of edge tones. Moreover, with the framework of LILt [26], which distinguishes between systematic and realizational sources of transfer, the L2 perception of post-focus tonal events can be scrutinized from both categorical and continuous perspectives

Another less researched aspect of cross-linguistic influences on L2 prosodic prominence is the interrogative modality. For Mandarin learners of English, this is of particular theoretical significance, as English prosodic prominence is conveyed through a low pitch accent in contrast to Mandarin focus [12]. Previous empirical studies have also found a falling bias in L1 Mandarin speakers at both lexical and post-lexical levels in processing L2 English [30,31,32,33]. For example, Ou [31] found that Taiwanese EFL learners perceived English stressed syllables cued with the high pitch accent (H*) better than those cued with the low pitch accent (L*). A similar trend has been observed in post-lexical prosody. L1 Mandarin speakers tend to process falling contours more effectively than rising ones. Rosenberg et al. [32] found that Mandarin speakers identified prosodic prominence in the intonational context of H*H-H% and L*H-H% less well than in H*L-L%. Zhang and Chen [33] had a similar finding that Mandarin speakers could perceive focus more accurately in statements than in questions. These studies demonstrate a clear advantage for L1 Mandarin speakers in processing L2 prominence within a falling tonal context. However, it remains to be determined to what extent this advantage is influenced by their L1 experience with a tone language, in which prosodic prominence is marked by pitch range expansion.

Apart from L1 influences, another factor in existing L2 speech models is the plasticity of L2 prosody development in late L2 learners. Given the ubiquitous L2 accent, many studies have explored what role L2 experience plays in the perception of interrogative focus prosody [1,34]. Some studies have found that L2 experience plays a significant role in L2 prosody perception, similar to its influence on the segmental development [35,36]. However, counterevidence has also been found that late learners have difficulties with L2 prosody, even after long exposure to the target language [34]. For example, Mennen [37] found that, despite having 12 to 35 years of L2 experience, advanced Dutch learners of Greek still encountered difficulties in peak alignment due to their L1 influences. She noted that, without perceptual data, it was challenging to determine whether the deviation resulted from a perceptual “deafness” to subtle cross-linguistic differences in peak alignment.

A relevant issue in methodology is how to define the length of L2 experience. It is generally believed that the longer the length of residence (LoR) in the target language environment, the less influence L1 has. However, once the LoR surpasses a certain threshold, the correlation between LoR and L1 influence no longer manifests [38]. It remains to be seen how long this threshold level should be before L2 speakers learn to mark prosodic interrogative focus efficiently.

### 1.3. The Current Study

Based on the comparative analysis of the three English tonal events in the AM model, it can be seen that the English focus interrogative tune, L*H-H%, provides an ideal window for research on L2 prosody development in Mandarin speakers. Firstly, the degree of cross-linguistic similarity ranges widely from being identical in boundary tones to being very different in pitch accents. Phrase accents are even more difficult to classify, for their variable domain size may pose different challenges for Mandarin speakers. In exploring how the continuum of L1-L2 similarity in focus prosody predicts the difficulty of L2 perception following the idea of SLM, more insights can be shed on the working mechanisms of cross-linguistic influences on L2 prosody perception. Secondly, L1 Mandarin speakers may be influenced by prosodic features at different tiers of the prosodic hierarchy, namely, the lexical and post-lexical levels. Examining how L1 Mandarin speakers perceive the low-rising pattern of English focus interrogative prosody may offer new insights into the mechanisms of cross-linguistic influence.

In this study, by adopting the AM model of intonation, we go beyond the perception of on-focus pitch accents and examine how L1 Mandarin prosody affects L2 English perception of pitch accents and edge tones, i.e., phrase accents and boundary tones, in the interrogative focus tune. By the choice of the interrogative focus prosody L*H-H%, we control for the semantic component in LILt. Cross-linguistic influences on L2 perception of L*H-H% are scrutinized from the perspectives of phonological categories and phonetic realization, which are, respectively, systematic and realizational dimensions. Specifically, we attempt to answer the following research questions: (1) To what extent do English–Mandarin cross-linguistic prosodic differences at phonological and phonetic levels affect the perception of English interrogative focus prosody, L*H-H%, by L1 Mandarin speakers? and (2) How does L2 experience influence the perception of English interrogative focus prosody by L1 Mandarin speakers? By addressing these questions, we aim to gain a deeper understanding of L2 focus prosody perception as an integral component of L2 phonological development and to offer insights for future pedagogical practices.

## 2. Materials and Methods

### 2.1. Participants

Eighteen Mandarin native speakers (mean age = 20.30, SD = 2.06) and eighteen English native speakers (mean age = 20.40, SD = 1.90) were recruited from the UCLA Psychology Subject Pool. They were all undergraduate students in their first or second year of study. All participants were rewarded 1 credit point for their participation. A language background survey adapted from LEAP-Q [39] was administered after the experiment. No participant reported any speech or hearing impairment. Their self-reported English proficiency level was comparable to IELTS 7.5 or TOEFL 100. The LoR of Mandarin speakers was calculated starting from their successive stay in the United States for longer than one month, except for the participant, M20, who moved to Canada at 11 years old. Considering the similar language background of these two countries, her LoR was calculated starting from that point. The data of M14 and M15, who were from Hong Kong, were later excluded from the analysis to avoid potential interference from their early L2 language exposure. For a similar reason, the data of E01, who was born in Singapore, was also excluded. The demographic information of participants is shown in Table 1.

### 2.2. Stimuli

Three tasks were designed to examine how the perception of pitch accents, phrase accents, and boundary tones in English interrogative focus prosody (L*H-H%) was affected by the results of the English–Mandarin comparison. Task 1 assessed whether listeners could detect prosodic prominence and identify the most prominent word. Task 2 evaluated whether listeners could understand the sentential focus correctly in question–answer pairs. Both tasks investigated the perception of on-focus L* in intonation contours. In addition to the on-focus pitch accent, we also examined how L1-L2 similarities/dissimilarities at both phonological and phonetic levels influenced English perception of the whole focus prosody contour by Mandarin speakers in Task 3.

Two American English speakers (one male and one female) were invited to record the stimulus materials (see Appendix A for all stimuli). They were both Ph.D. students in Linguistics and familiar with the ToBI-AME labeling system. Stimuli Set 1 was adapted from Rosenberg et al. [32]. By manipulating focus conditions (4 narrow focus positions + 1 broad focus condition) and intonation environments, 20 sentences were generated from two templates: “Roman women rarely marry” and “All men now run” (2 templates × 5 focus conditions × 2 intonation environments).

Stimuli Set 2 was composed of question–answer (QA) pairs, with the male voice asking questions and the female voice answering questions. Six Wh-questions and six yes–no question templates were first constructed. For each question template, the same sentence was provided, with only a narrow focus placed on the subject, verb, or object, respectively. There were 2 mismatched answers and 1 matched answer. To redress the imbalance in the chance level of matched and mismatched answers, we duplicated the matched answer to form 4 QA pairs for each question template. Out of each set of 4 QA pairs, 2 pairs were randomly chosen, and there was a total of 24 QA pairs in Task 2. Example QA pairs are shown below in (1) and (2):

(1) Q1: Did Mona **plan** the vacation?
  A1: No, Mona **ruined** the vacation. (Matched.)
  A2: No, Mona **ruined** the vacation. (Matched.)
  A3: No, **Mona** ruined the vacation. (Mismatched.)
  A4: No, Mona ruined the **vacation**. (Mismatched.)
(2) Q2: What did Anna eat?
  A1: Anna ate the **pie**. (Matched.)
  A2: Anna ate the **pie**. (Matched.)
  A3: Anna **ate** the pie. (Mismatched.)
  A4: **Anna** ate the pie. (Mismatched.)

Stimuli Set 3 was designed to test the perception of the whole intonational contour by asking listeners to judge the naturalness of 4 types of intonational tunes, including the canonical question tune (L*H-H%). As shown in Table 2, in addition to L*H-H%, the remaining 3 non-canonical tunes were generated by substituting L*, H-, and H%, respectively, in the canonical tune L*H-H%. Among them, Type 4 is of particular interest as it resembles Mandarin interrogative focus prosody in its phonetic realization. For each tune type, 5 communication situations were constructed. To reduce listeners’ fatigue, we randomly chose 3–5 situations for each tune type, and in total, 17 questions were presented to listeners (see Appendix A for details). To ensure the intelligibility of test stimuli, two labelers, who were blind to the purpose of the study, were asked to pre-test the materials for Task 1 and Task 2 and label the tunes used in Task 3 in the ToBI system.

### 2.3. Procedure

The experiment was conducted on the platform of *Appsobabble* developed by the UCLA Phonetic Laboratory. Listeners were seated before a computer screen in a sound-treated room with headphones, and they were asked to make choices based on the prompts shown on the screen and what they heard. It took participants around 25 min to finish all three tasks. Participants could choose to take a break between any 2 tasks. There was a trial block before each task, and listeners had a chance to ask questions before the experiment started.

In Task 1, listeners were instructed to choose the most prominent word in the sentence they heard by clicking the word number. They were asked to make decisions as quickly as possible. In Task 2, listeners were asked to decide if the prosody of the answer matched the question. The text of a QA pair would first appear on the screen, and in 500 ms, the recording would be played. Listeners then had to choose between the “Match” or “Mismatch” button by clicking the corresponding button. In Task 3, listeners were first asked to read carefully the situation and the question shown on the screen. Upon clicking the “NEXT” button, the recording would be played. Listeners were then supposed to rate the naturalness of the question on a scale from 1 to 5 based on what they read and heard, where 1 stands for the least natural and 5 the most. In all three tasks, the recordings could only be played once, but all tasks were self-paced by listeners through clicking the “NEXT” button.

### 2.4. Data Analysis

All responses were automatically collected by the experimental platform and analyzed using R.4.5.1 [40]. Data were all pre-checked for no missing values. For the results of Tasks 1 and 2, the binary dependent measure “accuracy” was fitted into binomial logistic regression models. For Task 1, “language group”, “intonation environment”, and their interaction were fixed factors. For Task 2, fixed factors were “language group”, “intonation environment”, “matchedness”, and their interactions. The results of Task 3 were analyzed using a Poisson logistic regression model, with “rating score” as the dependent variable and “language group,” “intonational tune,” and their interaction as fixed factors.

In addition, *Pearson* correlational analyses were conducted between the LoR of Mandarin speakers and their perception accuracy in Task 1 and Task 2. For Task 3 results, a *Pearson* correlational analysis was also performed between the rating and LoR of Mandarin speakers.

## 3. Results

### 3.1. On-Focus Pitch Accents

**Figure 3:**
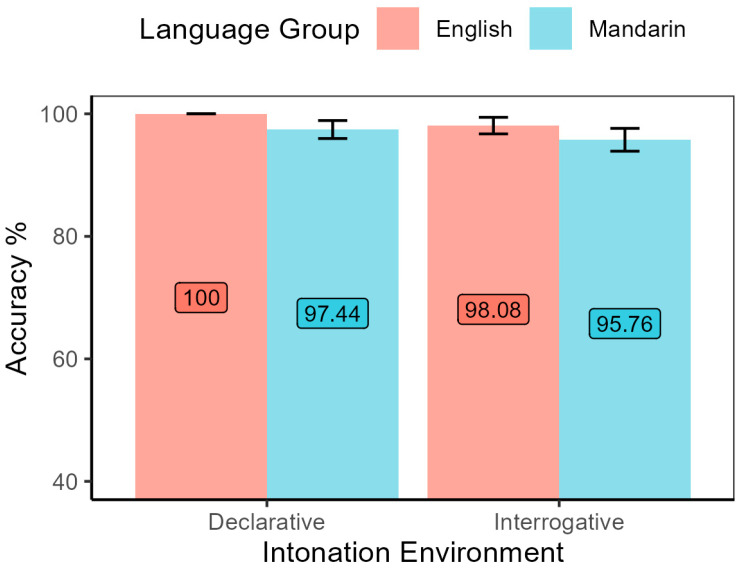
Accuracy of prominence detection by both language groups in declarative and interrogative environments.

The results of Task 1 show that both groups of listeners could tell the most prominent word from the other words with high accuracy in both statements and polar questions, despite the cross-linguistic difference in prosodic focus-marking. As shown in Figure 3, logistic regression found no significant effect of language group (*χ*^2^ = 3.23, *df* = 1, *p* = 0.07), nor main effect of intonation environment (*χ*^2^ = 1.67, *df* = 1, *p* = 0.20). There was no interaction effect as well (*χ*^2^ = 1.63, *df* = 1, *p* = 0.20). Participants from both language groups identified intonational prominence cued by both types of pitch accents, L* and H*, at a high accuracy rate. It could be seen that the categorical difference between declarative and interrogative modality was very small. Participants could easily identify the most important word in both types of sentence modality.

To examine whether the LoR of L2 listeners influenced their perception of pitch accents, a *Pearson* correlation analysis was conducted between the LoR of Mandarin speakers and their perceptual accuracy. However, no significant correlation was found (*r* = 0.14, *p* = 0.63).

**Figure 4:**
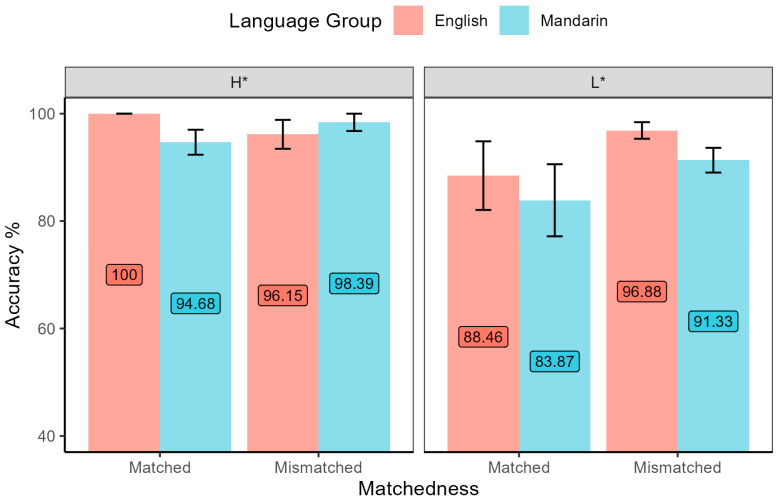
Accuracy of focus identification across conditions of matchedness and intonation environment by both language groups.

The results of Task 2 demonstrate how listeners identified focus in the interrogative intonation environments. A preliminary analysis found that four native English listeners missed Item 1, so the item was excluded from the statistical analysis. It can be seen from Figure 4 that both groups performed well in QA pairs with focus cued by H*, but for yes–no question pairs with L*, both groups identified focus with lower accuracy rates. The logistic regression results of Task 2 show the significant main effects of language group (*χ*^2^ = 4.96, *df* = 1, *p* = 0.03) and intonation type (*χ*^2^ = 7.15, *df* = 1, *p* = 0.01), as well as the three-way interaction of language group, intonation environment, and matchedness (*χ*^2^ = 5.50, *df* = 1, *p* = 0.02). Post-hoc tests found a marginally significant difference between language groups when in the mismatched interrogative condition (β = 1.08, *SE* = 0.59, *z* = 1.84, *p* = 0.07) but not in other conditions. In the focus identification task, the “L*” perception accuracy of Mandarin speakers was lower than that of English native speakers in conditions in which the intonation of questions and answers was mismatched.

Further *Pearson* correlational analysis was conducted between the LoR of Mandarin speakers and their perception accuracy in Task 2. There was no significant effect of LoR on the perception accuracy of Mandarin speakers (*r* = −0.24, *p* = 0.37).

### 3.2. Naturalness Judgment

**Figure 5:**
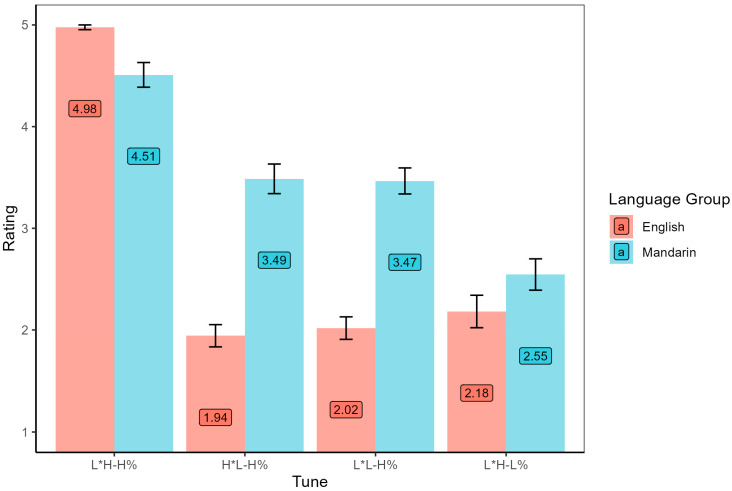
Rating scores of each intonation tune by both language groups in the interrogative intonation.

The results of Task 3 show ratings of each intonation tune in the interrogative intonation environment by both language groups. *Poisson* logistic regression was conducted with independent variables of language group and intonation tune type. As seen in Figure 5, there were significant main effects of language group (*χ*^2^ = 22.42, *df* = 1, *p* < 0.001) and intonation tune type (*χ*^2^ = 89.97, *df* = 3, *p* < 0.001). The interaction of these two factors was also significant (*χ*^2^ = 29.33, *df* = 3, *p* < 0.001). Further post-hoc comparisons found that the rating scores of English listeners were significantly lower than those of Mandarin listeners in the tune types H*L-H% (β = −0.58, *SE* = 0.12, *z* = −5.02, *p* < 0.001) and L*L-H% (β = −0.54, *SE* = 0.11, *z* = −4.72, *p* < 0.001). However, there was no difference between language groups in L*H-H% (β = 0.10, *SE* = 0.09, *z* = 1.06, *p* = 0.29) or L*H-L% (β = −0.15, *SE* = 0.15, *z* = −1.02, *p* = 0.31).

**Figure 6:**
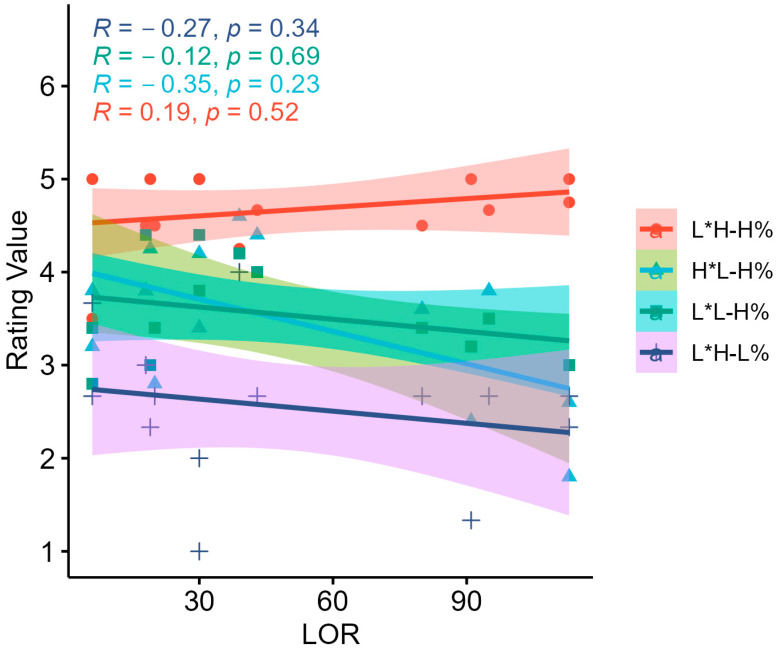
Pearson correlation between four intonation tune types and LoR.

*Pearson* correlation analyses were conducted to further test whether there were any correlations between the rating of each intonation tune type and LoR. As seen in Figure 6, no significant correlations were found between the LoR and any of the four intonation tune types.

## 4. Discussion

By employing three different tasks, i.e., prominence detection, focus identification, and naturalness judgment, we examined not only how listeners decoded the on-focus low pitch accent L* but also post-focus edge tones of phrase accents and boundary tones. In 4.1, we compared the difference in L2 perception of pitch accent L* between prominence detection and focus identification and discussed why non-canonical tune H*L-H% was rated as natural. In 4.2, we dealt with the perception of post-focus edge tones H% and H- by examining the rating results of non-canonical L*H-L% and L*L-H%. In 4.2, we discussed the role of L2 experience in the high rating of canonical L*H-H% and the poor performance in some non-canonical tunes. It can be seen that the degree of cross-linguistic similarities in pitch accents and edge tones could predict L2 perception differences to a large extent and that L2 experience interacts with cross-linguistic influences in a complicated way.

### 4.1. Cross-Linguistic Influences on the Perception of Pitch Accents

The results of three tasks show that L2 listeners performed well as native speakers in Task 1 but not in the other two tasks. As for cross-linguistic influences on the perception of pitch accent L*, L2 performance differences between Task 1 and the other two tasks can be explained by separating systematic (phonological) and realizational (phonetic) dimensions of L1 influences, as proposed in LILt [26]. On the one hand, the high accuracy in Task 1 prominence detection might be related to the perceptual hierarchy in phonetic realizations of prominence. Previous studies on L2 English perception of lexical stress by Mandarin speakers have shown that vowel quality and durational cues were dominant [41,42]. At the post-lexical level, although the pitch direction of Mandarin interrogative focus-marking is different from that of English, which marks the interrogative focus with a rising-tone L*, other acoustic correlates of prosodic salience are similar to English, with focused syllables longer in duration and higher in intensity [15]. Therefore, it is likely that listeners resort to more dominant acoustic cues of vowel quality and duration to identify the most prominent word, and they performed well both in declarative and interrogative intonational environments. Similar findings were also seen in Rosenberg et al. [32], in which Mandarin speakers were successful in identifying the most prominent word in L2 English cued by L*, even though some sentences did not make any sense. It seems that listeners could perform the task as if they were processing non-linguistic signals like those in Grabe et al. [29], where durational cues could have revealed more information on the prominence status than pitch height.

On the other hand, poor performance in focus identification and naturalness judgment tasks showed that these cross-linguistic similarities in phonetic dimensions might not be adequate when meaning processing was involved. Phonological differences may play a more important role in the perception of L*. In Task 2, the results show that L2 speakers tended to perceive L* less accurately in mismatched cases, but only with marginal significance. It was likely that meaningful materials in the interrogative environment posed more challenges for them as higher-order cognitive processes were involved. In Baker [35], a similar pattern was also seen, where L2 listeners tended to perform worse in the focus understanding task than in prominence detection. Ortega-Llebaria and Colantoni [43] also found that cross-linguistic influences on intonation were more obvious when listeners had access to meanings. In our study, it seems that though Mandarin speakers were proficient in their detection of the nuclear pitch accent L* in Task 1, they were less able to map it into the meaning of focus in Task 2. However, due to the limited number of participants in our study and the marginal significance, further research is needed to confirm this pattern.

Apart from L2 inaccuracies in Task 2 focus identification, the rating results of H*L-H% in Task 3 natural judgment could also be attributed to the phonological influence of L1 prosodic encoding on the perception of L*. In contrast to English native speakers, L2 listeners incorrectly accepted non-canonical H*L-H% as natural as L*H-H%, the canonical English interrogative tune. This deviation could be attributed to the transfer of L1 interrogative focus prosody. In Mandarin, focus meanings are conveyed through pitch range expansion on-focus and pitch range compression post-focus, with all lexical tone shape preserved, as mentioned in Section 1.1. This on-focus pitch range expansion is more likely to be interpreted by Mandarin speakers to be a high pitch target similar to H* rather than a low tone target in L*, as evidenced in Liu et al. [44], who found the perceptual threshold for focus was three semitones higher than the baseline. In addition, as demonstrated in previous studies on prosodic prominence at the lexical and post-lexical levels [30,31,45], Mandarin speakers tended to associate prominence with a high falling pitch pattern. This could also account for the high rating of H*L-H% by L1 Mandarin speakers in that “H*L-” forms a falling pattern, but “L*H-” forms a rising tone. In this sense, H*L-H% is among the most natural candidates for Mandarin speakers to convey focus meaning. Further research could be conducted to determine whether this over-tolerance with H*L-H% can account for L2 production deviations in focus prosody.

### 4.2. Cross-Linguistic Influences on Edge Tone Perception

To see how L2 perception of post-focus edge tones was affected by L1 prosodic features, we compared the ratings of the canonical interrogative focus prosody, L*H-H%, to that of each non-canonical tune, respectively, i.e., L*H-L%, L*L-H%, and H*L-H%.

An obvious pattern in the perception of boundary tones could be seen from the rating of L*H-L%, which both Mandarin and English speakers unanimously rated as unnatural. The correct rejection of L*H-L% by Mandarin speakers can be largely attributed to the form–function equivalence in Mandarin and English boundary tones. In comparison to the canonical tune L*H-H%, which Mandarin speakers also accepted as natural, the only difference between the two intonational tunes was in the boundary tone, (L*H-) H% vs. (L*H-) L%. It was then safe to infer that Mandarin speakers reject L*H-L% based on the boundary tone, L%, which was used in Mandarin declaratives. As pointed out in Lin [16,46], the key prosodic difference between unmarked yes–no questions and statements in Mandarin is the difference in boundary tone. In Mandarin, H% is associated with yes–no questions without interrogative particles such as “ma”. When asked to rate the naturalness of an intonational tune, it was highly possible that Mandarin speakers resorted to their L1 prosodic marking of interrogativity. As pointed out in LIIt, following the “equivalence classification” of segments proposed in SLM [24,47], where there is no difference, there is no cross-linguistic influence. At the suprasegmental level, the perception of boundary tones might have worked in a similar way, given such a complete match in their form and function between Mandarin and English. In this sense, L*H-L%, with its low boundary tone L%, was obviously not a good choice for them.

In comparison, cross-linguistic influences on phrase accents were more elusive in that the deviation in ratings of the non-canonical tune L*L-H% by Mandarin speakers could involve the perception of phrase accent “L-” and boundary tone “H%”. Firstly, for Mandarin speakers, “H%” in L*L-H% sounded natural, as the speaker intended to seek confirmation with an unmarked polar question. Since Mandarin speakers associated the meaning of interrogativity with the final high boundary tone H% [46,48,49], it is possible that L*L-H% sounded natural to Mandarin speakers, as the whole pitch contour was rising, and that “L-” in the tune was totally overlooked. To figure out exactly which category affects L2 listeners, future studies could be designed using the gating method, exemplified by Shang et al. [50].

Another reason that Mandarin listeners were not able to reject L*L-H% might lie in the fact that Mandarin speakers could not identify the contrast between the high and low phrase accents (“H-” vs. “L-”), for it was the only difference between the two tunes L*L-H% and L*H-H%. First, the lack of the phonological category of phrase accents in Mandarin could function in a similar way to the stress epenthesis in French speakers [51,52]. French speakers could not tell the difference between different positions of L2 lexical stress, for stress is not linguistically relevant in French lexical decisions. In the same vein, since there is no corresponding phonological category of phrase accent in Mandarin, the contrast between the high and low phrase accents (“H-” vs. “L-”) might not be linguistically relevant for Mandarin speakers.

In addition, phrase accents could pose more challenges to L2 listeners due to their less discernible linguistic features. Unlike pitch accents or boundary tones, the phonological domain of a phrase accent is unfixed, ranging from a syllable to a phrase. English intonational tunes work at an autosegmental layer, consisting of intonational events with variable sizes of tone-bearing units, floating on the layer of syllable/phonological words [6]. However, Mandarin pitch contours are densely specified by lexical tones. When learning an intonation language, in which tones are only sparsely specified, Mandarin speakers would have a less clear idea of what a post-lexical contour inventory should be like. With the interference of the existing L1 prosody structure, Mandarin speakers were less likely to perceive such a delicate contrast between high and low phrase accents (“H-” vs. “L-”). Another possibility is related to the unmarked form of the low pitch in “L-”. As pointed out by Evans [53], a raised F0 is mostly used to attract the attention of listeners. In L*L-H%, the attention of listeners might be aroused with such a low and flat contour, unless the pitch changes for focus-marking. It was then unsurprising for Mandarin speakers to accept L*L-H% as a good option.

In L2 development of prosody, it is, therefore, particularly important to take the whole system of phonological structure into consideration. Unlike segments that have a comparable domain within a syllable, suprasegmental features may function in more varied and undefined domains.

In summary, the results of pitch accents and boundary tones show that the perception of interrogative focus prosody was not only built up with acoustic signals but, more importantly, related to the expectations of listeners on the relationship between phonological structure and prosodic cues. As pointed out by Bohn [54], L1 influences do not change the perception of auditory signals but only mold the noticing of listeners, which is usually determined by L1 phonological encoding. Bishop et al. [55] also found that phrase-level prominence perception can be complicated, being affected by phonology (pitch accent status and pitch accent type) and signal-based and signal-extrinsic factors. In this sense, previous knowledge based on L1 prosodic features could constrain or impede L2 perception of focus prosody. Phonological encoding required in high-order cognitive activities is more subject to L1 influences than auditory mechanisms. We can conclude that the L2 perception of interrogative focus prosody, which involves greater cognitive challenges, is likely to be influenced by existing L1 features.

### 4.3. Role of L2 Experience in the Acquisition of Interrogative Focus Prosody

In addition to the crosslinguistic influences, this study also explored the issue of the perceptual plasticity of late L2 learners by exploring the role of L2 experience in the development of interrogative focus prosody perception. As shown in the correlational results, we found that none of the task performance was related to the LoR of Mandarin speakers. At least three aspects of L2 performance were of particular interest. First, given that L2 performance was comparable in native speakers in both Task 1 and Task 2, it is possible that for the perception of on-focus pitch accents, even LoR as short as 0.42 years might have well surpassed a threshold, as suggested by Piske et al. [38]. L2 speakers might well have been able to identify the prominent part, be it cued by H* or L*.

Second, despite its difference from Mandarin prosody, English canonical interrogative focus prosody L*H-H% could be accurately perceived. This could be related to the amount of L2 experience in our study. Studies on segmental learning found that the burst effects of LoR were usually seen within the first year of immigration [47,56]. In intonation perception, Grabe et al. [29] found that even Mandarin speakers who were based in their native country could already perceive L*H-H% well. In our study, the minimum LoR of participants was 5 months, and their average age of learning was 7.75 years old. In this sense, it was not surprising that their perception of L*H-H% was good enough, given their L2 experience.

In spite of the success in L2 perception of pitch accents and L*H-H%, no evidence was found between extended LoR and L2 success in rejecting non-canonical interrogative focus tunes. In our study, the average LoR was 3.65 years, ranging from 0.42 to 9.33 years. However, Mandarin native speakers were still different from English native speakers in their rating of the non-canonical tunes L*L-H% and H*L-H%. This deviation is consistent with a widely accepted observation that L2 prosody is a thorny part in L2 phonology acquisition ([54]; see Mennen & de Leeuw [1] for a review). It is also consistent with previous studies, such as Trofimovich and Baker [36] and Huang and Jun [57], that there might be variations in different aspects of L2 development in prosody. For example, in our study, the perception of pitch accents or the tune of L*H-H% was learned early in L2 development. However, the less prominent tunes posed a challenge for L2 learners, even with a decade of LoR.

Apart from its length, the exact nature of L2 experience is also relevant in that the frequency of target language use and the quality of language input may vary greatly, as pointed out by Trofimovich and Baker [36]. In our study, the language background survey showed that in terms of daily use of English, fewer than 20% of participants reported more than 4 h, and half of them used English between 2 and 4 h, and the rest of them reported using only about 1 h. In addition, very few participants reported that they had been involved in an explicit teaching program targeted at L2 pronunciation. This lack of contact with the target language might, in a way, offset the possible effect of their long LoR, thus contributing to the poor performance in L2 perception.

In summary, one limitation of the present study lies in the relatively small sample size and a wide range of LoR from 0.42 to 9.42 years in the L2 group. To confirm these preliminary findings and understand the nature of L2 experience further, future work could include larger, more representative samples with a wider range of variables, like age of learning or age of arrival. In addition to these indices on the length of language experience, closer examination of the L2 exposure quality or language use could also be useful.

## 5. Conclusions

This study investigated how Mandarin native speakers perceive English interrogative focus prosody L*H-H% by examining both on-focus and post-focus intonational categories. It can be seen that Mandarin speakers process the rising tone L* differently from native English speakers, despite its phonetic similarity to Mandarin lexical tones. The perception results of on-focus pitch accents confirm that the influence of L1 functions not at the acoustic processing level but at the linguistic level. As for the perception of post-focus intonational categories, the degree of cross-linguistic similarities was found to be relevant in accounting for the rating differences. All these findings were discussed in the framework of LILt.

In comparison to the L1 influence, language experience proved less useful in improving L2 perception of English prosody. There was no clear correlation between the LoR of Mandarin speakers and their perception accuracy. In the future, instead of cross-sectional design, longitudinal studies on the influences of language experience on L2 prosody development could be conducted to further our understanding of the L2 learning process. The significance of such research lies in its potential to inform both theoretical models of language acquisition and practical applications for language teaching. By identifying the key factors that influence L2 prosody development, educators could better tailor instructional strategies to the needs of learners, promoting more effective and individualized teaching practices.

## Figures and Tables

**Table 1:** Demographic information of participants by group.

Group	*n*	Age Mean (SD)	LoR Mean (SD)
Mandarin native	18	20.25 (2.08)	3.66 (2.83)
English native	18	20.41 (1.91)	/

**Table 2:** Intonation tune types in Task 3.

No.	Pitch Accents	Phrase Accents	Boundary Tones	Canonical
1	L*	H-	H%	Yes
2	L*	H-	L%	No
3	L*	L-	H%	No
4	H*	L-	H%	No

## Data Availability

The original data presented in the study are openly available at https://osf.io/w2qvc**/** (accessed on 31 July 2025).

## Appendix A. Experimental Materials

The three tasks designed for the experiment are prominence detection, focus identification, and naturalness judgment. The experimental materials for all three tasks are as follows:

**Table A1:** Task 1: Prominence detection task.

1	Roman women rarely marry.
2	**Ro**man women rarely marry.
3	Roman **wo**men rarely marry.
4	Roman women **rare**ly marry.
5	Roman women rarely **mar**ry.
6	Roman women rarely marry?
7	**Ro**man women rarely marry?
8	Roman **wo**men rarely marry?
9	Roman women **rare**ly marry?
10	Roman women rarely **mar**ry?
11	All men now run.
12	**All** men now run.
13	All **men** now run.
14	All men **now** run.
15	All men now **run**.
16	All men now run?
17	**All** men now run?
18	All **men** now run?
19	All men **now** run?
20	All men now **run**?

**Table A2:** Task 2: Focus identification task.

1	Match	1	s	Who ate the pie?	**Anna** ate the pie.
	Match	2	s	Who ate the pie?	**Anna** ate the pie.
	Mismatch	3	s	Who ate the pie?	Anna **ate** the pie.
	Mismatch	4	s	Who ate the pie?	Anna ate the **pie**.
2	Match	5	v	What did Anna do with the pie?	Anna **ate** the pie.
	Match	6	v	What did Anna do with the pie?	Anna **ate** the pie.
	Mismatch	7	v	What did Anna do with the pie?	**Anna** ate the pie.
	Mismatch	8	v	What did Anna do with the pie?	Anna ate the **pie**.
3	Match	9	o	What did Anna eat?	Anna ate the **pie**.
	Match	10	o	What did Anna eat?	Anna ate the **pie**.
	Mismatch	11	o	What did Anna eat?	Anna **ate** the pie.
	Mismatch	12	o	What did Anna eat?	**Anna** ate the pie.
4	Match	13	s	Who broke the vase?	**Lenny** broke the vase.
	Match	14	s	Who broke the vase?	**Lenny** broke the vase.
	Mismatch	15	s	Who broke the vase?	Lenny **broke** the vase.
	Mismatch	16	s	Who broke the vase?	Lenny broke the **vase**.
5	Match	17	v	What did Lenny do to the vase?	Lenny **broke** the vase.
	Match	18	v	What did Lenny do to the vase?	Lenny **broke** the vase.
	Mismatch	19	v	What did Lenny do to the vase?	**Lenny** broke the vase.
	Mismatch	20	v	What did Lenny do to the vase?	Lenny broke the **vase**.
6	Match	21	o	What did Lenny break?	Lenny broke the **vase**.
	Match	22	o	What did Lenny break?	Lenny broke the **vase**.
	Mismatch	23	o	What did Lenny break?	Lenny **broke** the vase.
	Mismatch	24	o	What did Lenny break?	**Lenny** broke the vase.
7	Match	25	v	Did Mona PLAN the vacation?	No, Mona **ruined** the vacation.
	Match	26	v	Did Mona PLAN the vacation?	No, Mona **ruined** the vacation.
	Mismatch	27	v	Did Mona PLAN the vacation?	No, **Mona** ruined the vacation.
	Mismatch	28	v	Did Mona PLAN the vacation?	No, Mona ruined the **vacation**.
8	Match	29	s	Did MARY ruin the vacation?	No, **Mona** ruined the vacation.
	Match	30	s	Did MARY ruin the vacation?	No, **Mona** ruined the vacation.
	Mismatch	31	s	Did MARY ruin the vacation?	No, Mona **ruined** the vacation.
	Mismatch	32	s	Did MARY ruin the vacation?	No, Mona ruined the **vacation**.
9	Match	33	o	Did Mona ruin the GATHERING?	No, Mona ruined the **vacation**.
	Match	34	o	Did Mona ruin the GATHERING?	No, Mona ruined the **vacation**.
	Mismatch	35	o	Did Mona ruin the GATHERING?	No, **Mona** ruined the vacation.
	Mismatch	36	o	Did Mona ruin the GATHERING?	No, Mona **ruined** the vacation.
10	Match	37	s	Did RONAN call the agent?	No, **Laura** called the agent.
	Match	38	s	Did RONAN call the agent?	No, **Laura** called the agent.
	Mismatch	39	s	Did RONAN call the agent?	No, Laura **called** the agent.
	Mismatch	40	s	Did RONAN call the agent?	No, Laura called the **agent**.
11	Match	41	v	Did Laura EMAIL the agent?	No, Laura **called** the agent.
	Match	42	v	Did Laura EMAIL the agent?	No, Laura **called** the agent.
	Mismatch	43	v	Did Laura EMAIL the agent?	No, **Laura** called the agent.
	Mismatch	44	v	Did Laura EMAIL the agent?	No, Laura called the **agent**.
12	Match	45	o	Did Laura call the MANAGER?	No, Laura called the **agent**.
	Match	46	o	Did Laura call the MANAGER?	No, Laura called the **agent**.
	Mismatch	47	o	Did Laura call the MANAGER?	No, **Laura** called the agent.
	Mismatch	48	o	Did Laura cald the MANAGER?	No, Laura **called** the agent.

**Table A3:** Task 3: Naturalness judgment task.

	Situation		Materials		
1	The teacher knows that ***Mona*** finished her homework on time, but she wants to know if ***Anna*** also finished her homework on time. She says,	1	LHH	I know Mona finished her homework on time, but	did ANNA finish her homework on time?
		2	LHL	I know Mona finished her homework on time, but	did ANNA finish her homework on time?
		3	LLH	I know Mona finished her homework on time, but	did ANNA finish her homework on time?
		4	HLH	I know Mona finished her homework on time, but	did ANNA finish her homework on time?
2	The manager knows that ***Joana*** went to New York by train, but now she wants to know if ***Emma*** also went to New York by train. She says,	5	LHH	I know Joana went to New York by train, but	did EMMA go to New York by train?
		6	LHL	I know Joana went to New York by train, but	did EMMA go to New York by train?
		7	LLH	I know Joana went to New York by train, but	did EMMA go to New York by train?
		8	HLH	I know Joana went to New York by train, but	did EMMA go to New York by train?
3	The chef knows ***Leo*** made the cupcake by himself, but she wants to know if ***Manny*** also made the cupcake by himself. She says,	9	LHH	I know Leo made the cupcake by himself, but	did Manny make the cupcake by himself?
		10	LHL	I know Leo made the cupcake by himself, but	did Manny make the cupcake by himself?
		11	LLH	I know Leo made the cupcake by himself, but	did Manny make the cupcake by himself?
		12	HLH	I know Leo made the cupcake by himself, but	did Manny make the cupcake by himself?
4	Mum knows ***Jason*** passed the drive test last week, but she wants to know if ***Nolan*** also passed the drive test last week. She says,	13	LHH	I know Jason passed the driving test last week, but	did Nolan pass the driving test last week?
		14	LHL	I know Jason passed the driving test last week, but	did Nolan pass the driving test last week?
		15	LLH	I know Jason passed the driving test last week, but	did Nolan pass the driving test last week?
		16	HLH	I know Jason passed the driving test last week, but	did Nolan pass the driving test last week?
5	The agent knows that ***Lily*** hit the road sign by mistake, but she wants to know if ***Ella*** also hit it by mistake. She says,	17	LHH	I know Lily hit the road sign by mistake, but	did Ella hit the road sign by mistake?
		18	LHL	I know Lily hit the road sign by mistake, but	did Ella hit the road sign by mistake?
		19	LLH	I know Lily hit the road sign by mistake, but	did Ella hit the road sign by mistake?
		20	HLH	I know Lily hit the road sign by mistake, but	did Ella hit the road sign by mistake?
